# Supplementary material for: Trigonally Distorted Hexacoordinate Co(II) Single-Ion Magnets
Source: Materials (Basel). 2022 Jan 29;15(3):1064. doi: 10.3390/ma15031064 (PMC8839918; doi:10.3390/ma15031064)
Supplement: Supplementary file 1 [file materials-15-01064-s001.zip › materials-1518178-supplementary.pdf]

materials

Supplementary

Trigonally Distorted Hexacoordinate Co(II) Single-Ion Magnets

Ivan Nemec [1,2,*], Ondrej F. Fellner [1], Berenika Indruchova [1], and Radovan Herchel [1]

[1] Department of Inorganic Chemistry, Faculty of Science, Palacký University, 17. listopadu 12, 771 46 Olomouc, Czech Republic; ondrej.fellner01@upol.cz (O.F.F.); berenika.indruchova01@upol.cz (B.I.); radovan.herchel@upol.cz (R.H.)
[2] Central European Institute of Technology, Brno University of Technology, Purkynova 123, 61200 Brno, Czech Republic
* Correspondence: ivan.nemec@upol.cz

Contents

Figure S1. XRPD data for 1 .... S2

Figure S2. XRPD data for 2 .... S2

Figure S3. XRPD data for 3 .... S3

Figure S4. A perspective view on a 2D network S4

Figure S5. A three-dimensional plot of molar magnetization S6

Figure S6. In-phase χ_{real} and out-of-phase χ_{imag} molar susceptibilities for 1 .... S6

Figure S7. In-phase χ_{real} and out-of-phase χ_{imag} molar susceptibilities for 2 .... S7

Figure S8. In-phase χ_{real} and out-of-phase χ_{imag} molar susceptibilities for 3 .... S8

Figure S9. The low-lying ligand-field multiplets originating from the $^4T_{1g}$ state of 1-3 .... S9

Figure S10. The analysis of the magnetic data calculated from CASSCF/NEVPT2 calculations S10

Table S1. The results of SHAPE calculation for 1-3 .... S10

Table S2. Parameters of one-component Debye's model for 1 .... S11

Table S3. Parameters of one-component Debye's model for 2 .... S11

Table S4. Parameters of one-component Debye's model for 3 .... S12

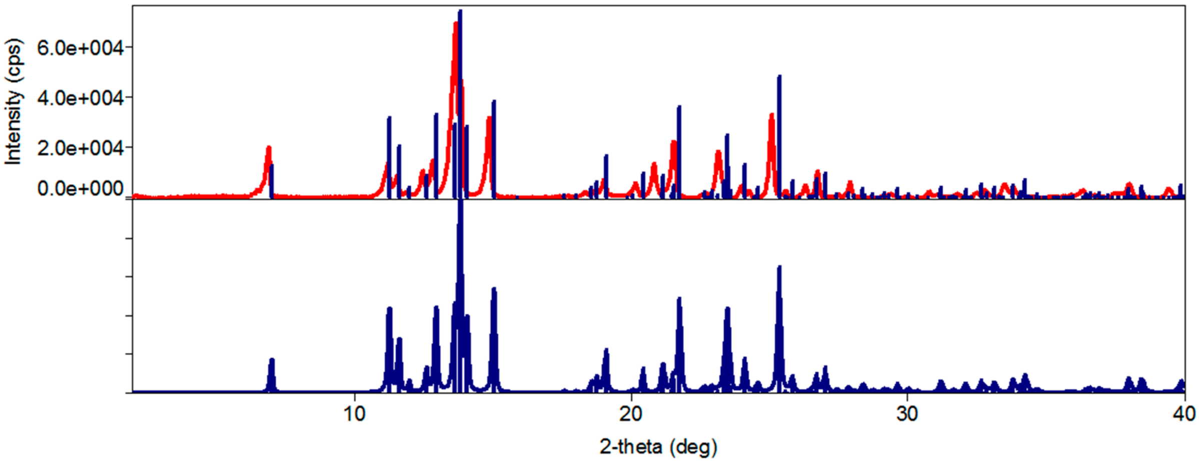

Figure S1. XRPD data for 1 (λ = 1.54056 Å); Red lines: an experimental diffraction pattern, blue lines: a pattern calculated from the single-crystal structure.

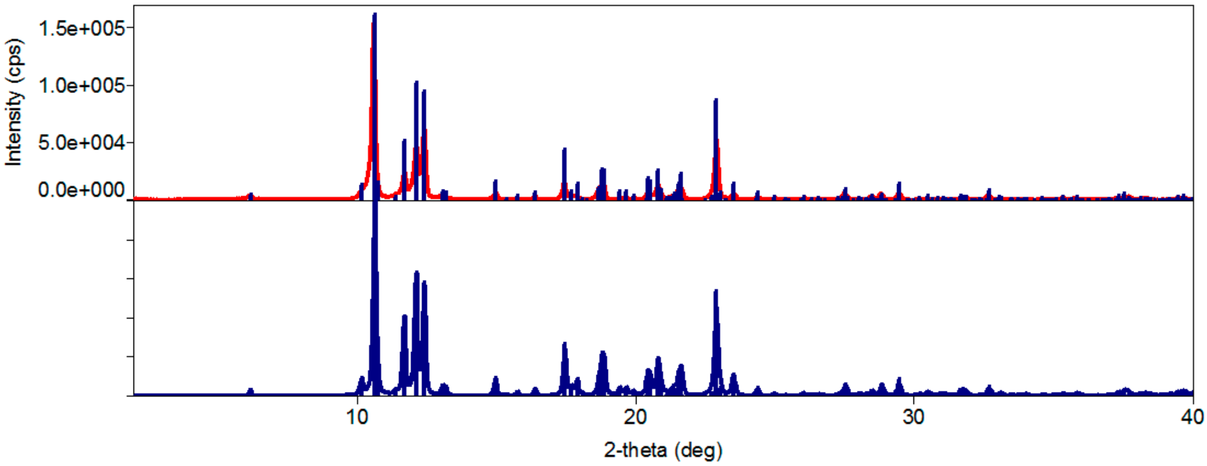

Figure S2. XRPD data for 2 (λ = 1.54056 Å); Red lines: an experimental diffraction pattern, blue lines: a pattern calculated from the single-crystal structure.

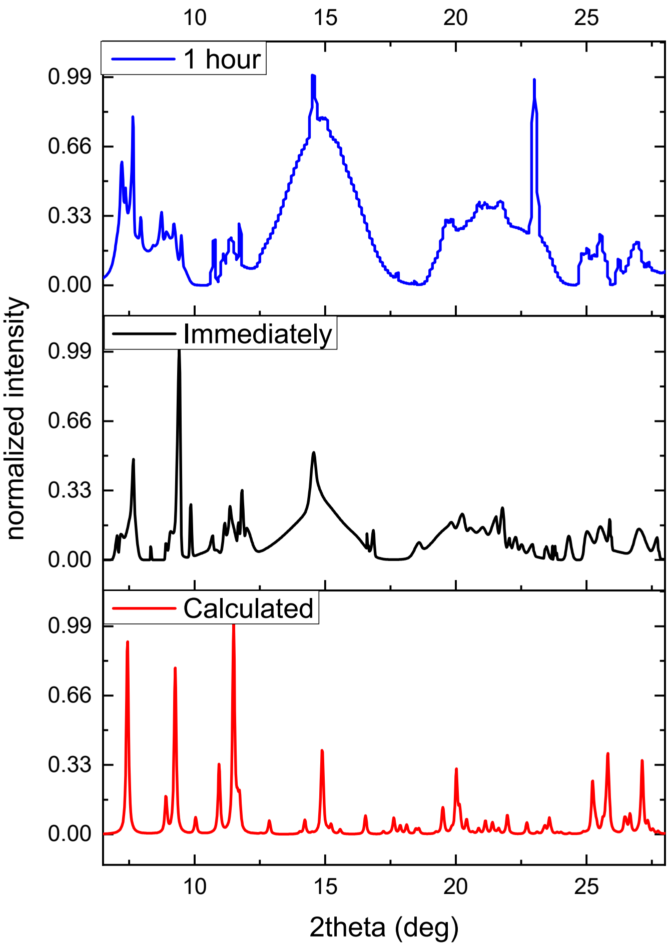

Figure S3. XRPD data for 3 (λ = 1.54056 Å). Crystals were immersed in a crystallographic oil and grinded. Measurement (scan speed 10°/min) done immediately after isolating from the mother liquor (black line), 1h later (blue line). Red lines: a pattern calculated from the single-crystal structure of **3**.

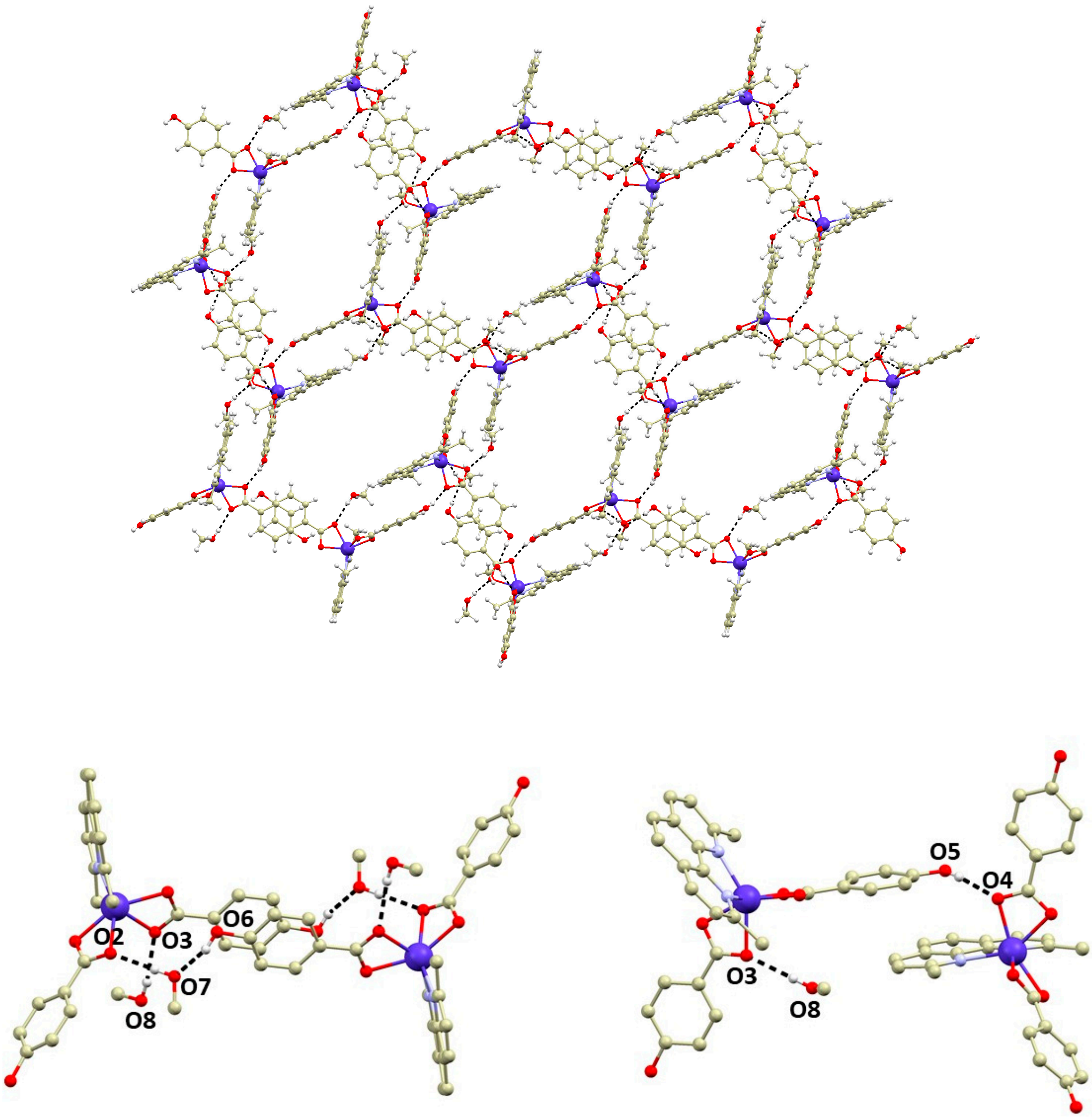

Figure S4. A perspective view on a 2D network formed by O–H···O hydrogen bonding (*above*). Perspective views on two fragments from 2D network (*below*). Hydrogen bonds were depicted with black dashed lines. Hydrogen atoms in the figure below were omitted for clarity (except for those involved in hydrogen bonding). Color code: Carbon (brown), Cobalt (dark blue), Nitrogen (light blue), Oxygen (red). Selected donor···acceptor distances (in Å): *d*(O5···O4) = 2.649(5), *d*(O6···O7) = 2.667(7), *d*(O7···O2) = 2.702(6), *d*(O8···O3) = 2.846(7).

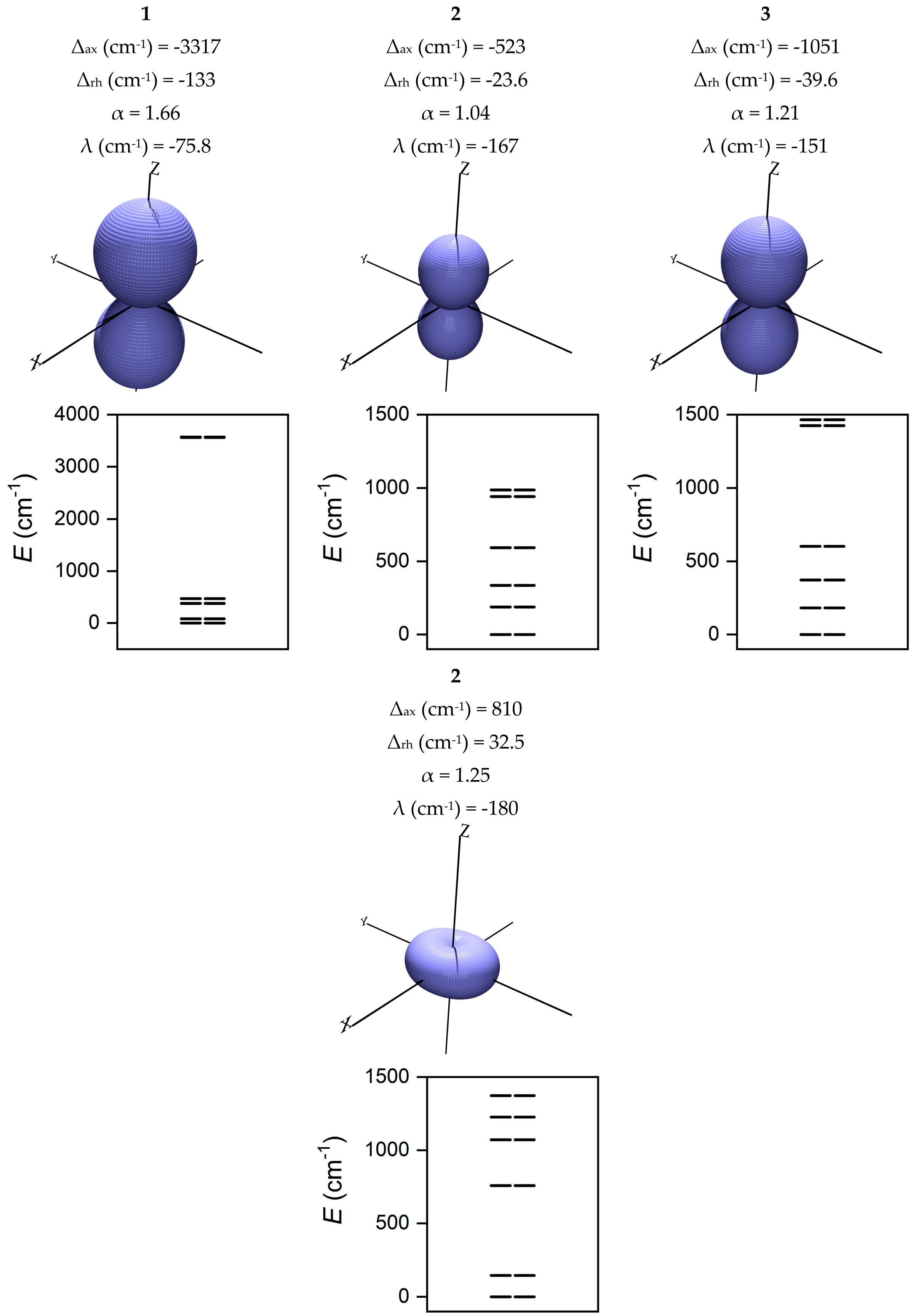

1
Δax (cm-1) = -3317
Δrh (cm-1) = -133
α = 1.66
λ (cm-1) = -75.8
Z
Y
X
4000
3000
2000
1000
0
E (cm-1)
2
Δax (cm-1) = -523
Δrh (cm-1) = -23.6
α = 1.04
λ (cm-1) = -167
Z
Y
X
1500
1000
500
0
E (cm-1)
3
Δax (cm-1) = -1051
Δrh (cm-1) = -39.6
α = 1.21
λ (cm-1) = -151
Z
Y
X
1500
1000
500
0
E (cm-1)
2
Δax (cm-1) = 810
Δrh (cm-1) = 32.5
α = 1.25
λ (cm-1) = -180
Z
Y
X
1500
1000
500
0
E (cm-1)

Figure S5. A three-dimensional plot of molar magnetization (T = 2 K, B = 1 T) calculated with the fitted parameters of **1-3** from the experimental magnetic data and the respective energy levels in the zero magnetic field.

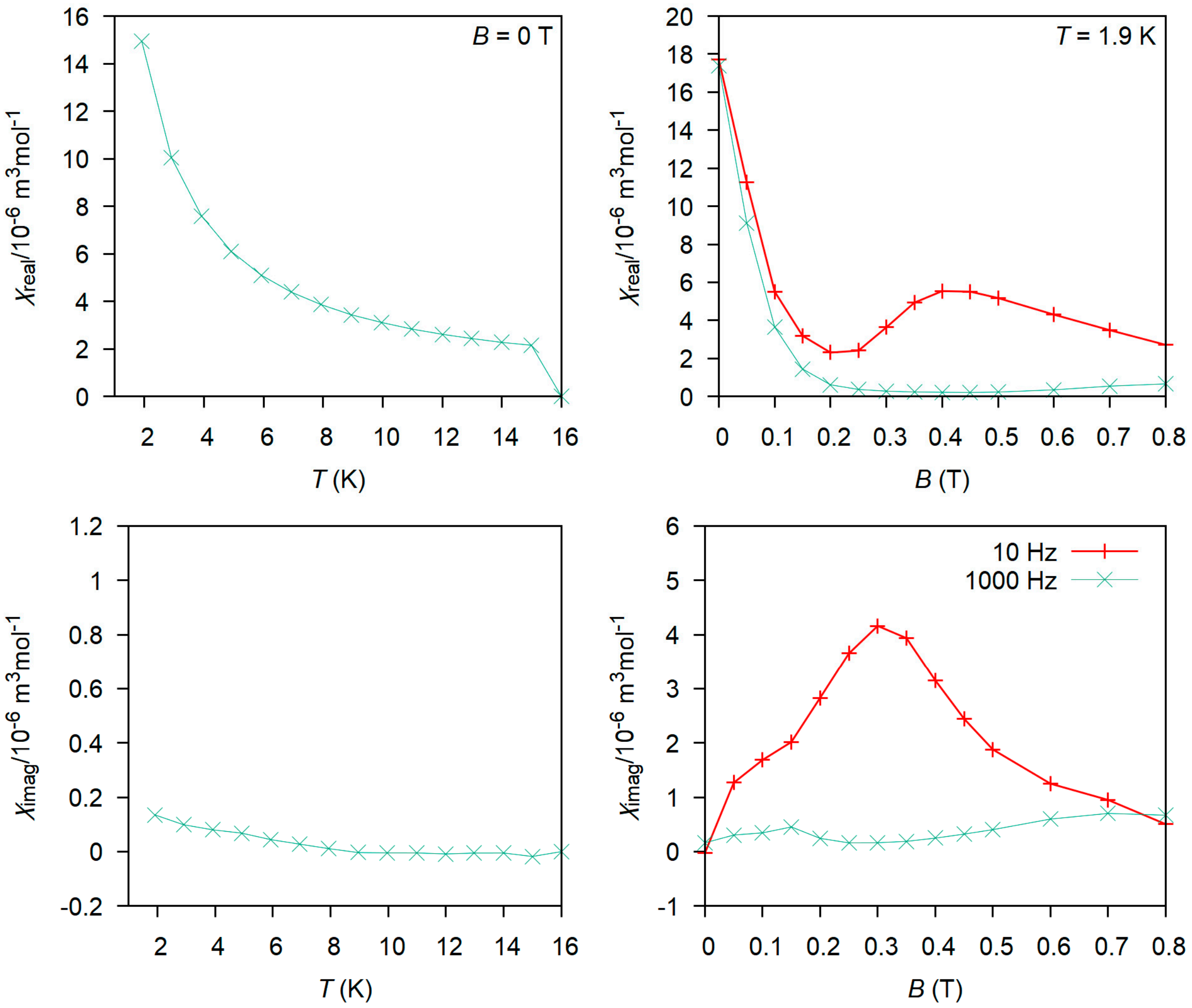

Figure S6. In-phase χ_{real} and out-of-phase χ_{imag} molar susceptibilities for 1 at the zero static magnetic field and in a non-zero static field. Lines serve as guides for the eyes.

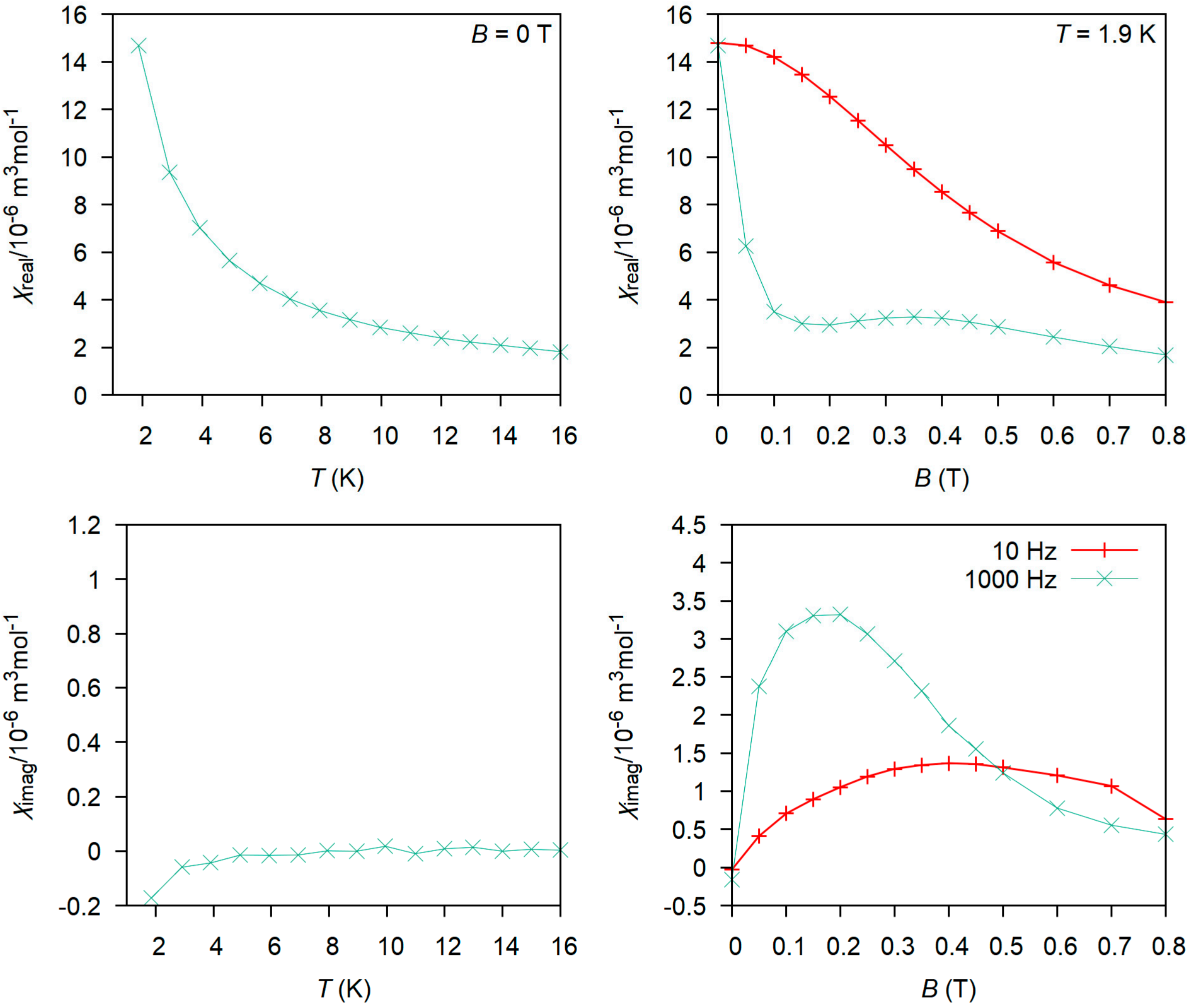

Figure S7. In-phase χ_{real} and out-of-phase χ_{imag} molar susceptibilities for 2 at the zero static magnetic field and in a non-zero static field. Lines serve as guides for the eyes.

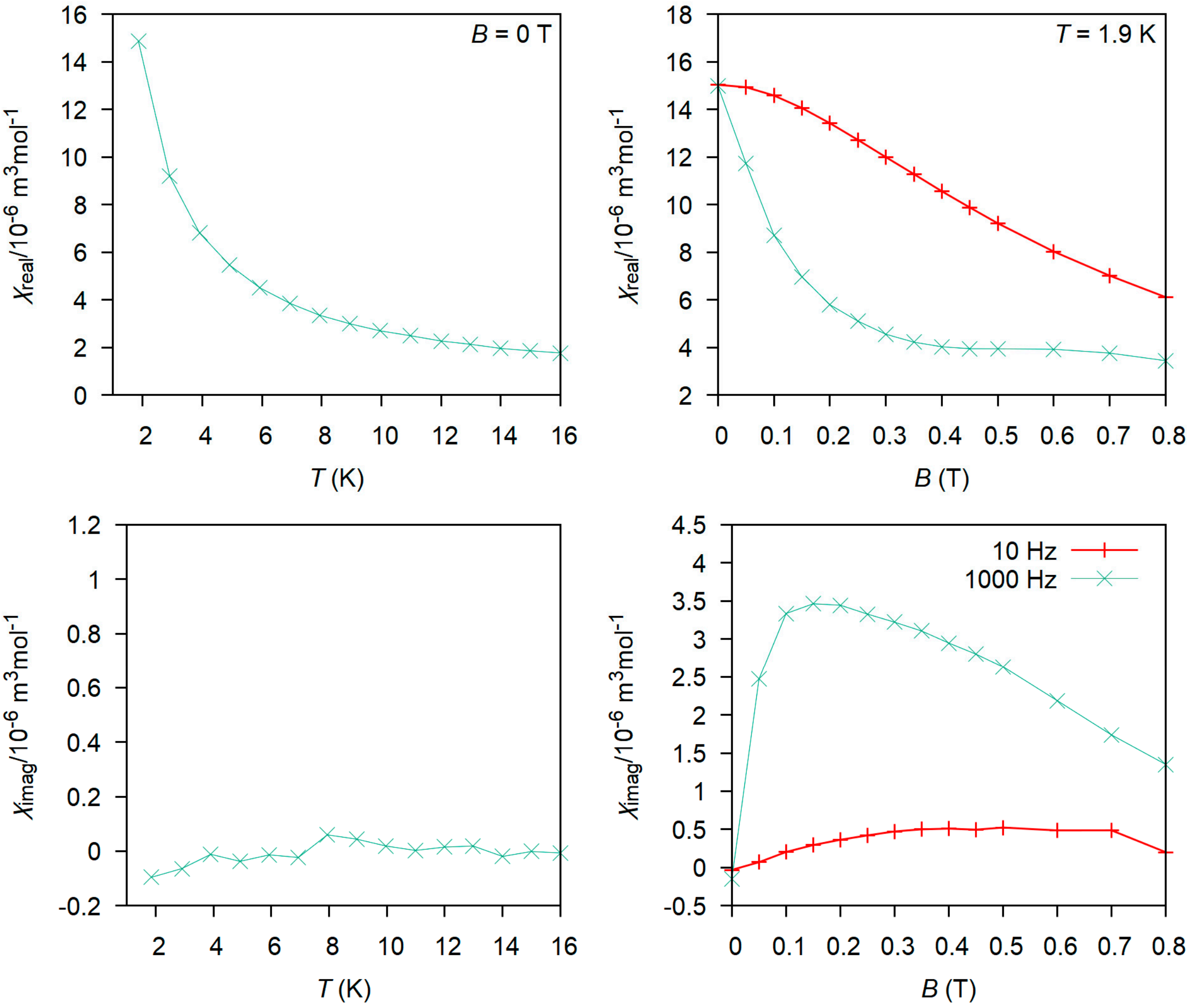

Figure S8. In-phase χ_{real} and out-of-phase χ_{imag} molar susceptibilities for 3 at the zero static magnetic field and in a non-zero static field. Lines serve as guides for the eyes.

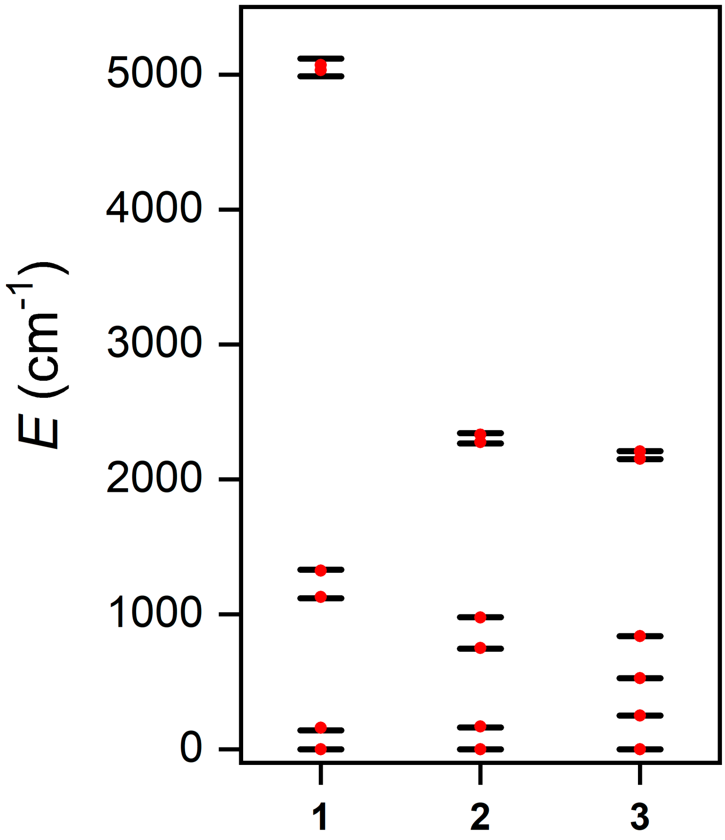

Figure S9. The low-lying ligand-field multiplets originating from the $^4T_{1g}$ state of 1-3 **resulting** from CASSCF/NEVPT2 calculations – black lines. The fitted data with the L-S Hamiltonian in Eq.1 with the values of $\alpha\lambda$, Δ_{ax} and Δ_{rh} listed in Table 2.

1

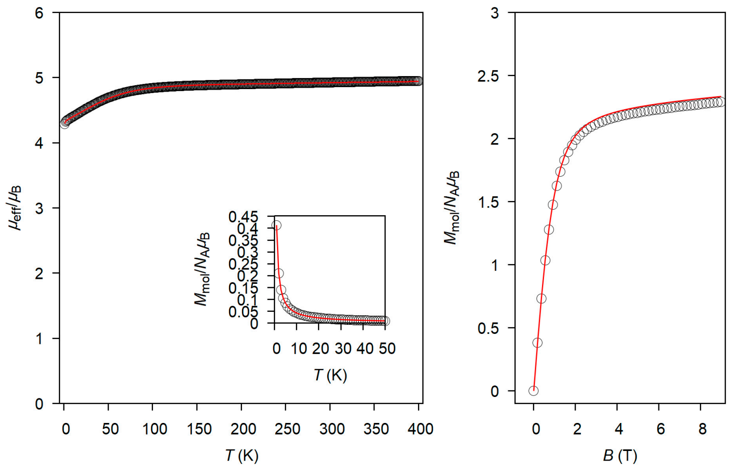

2

3

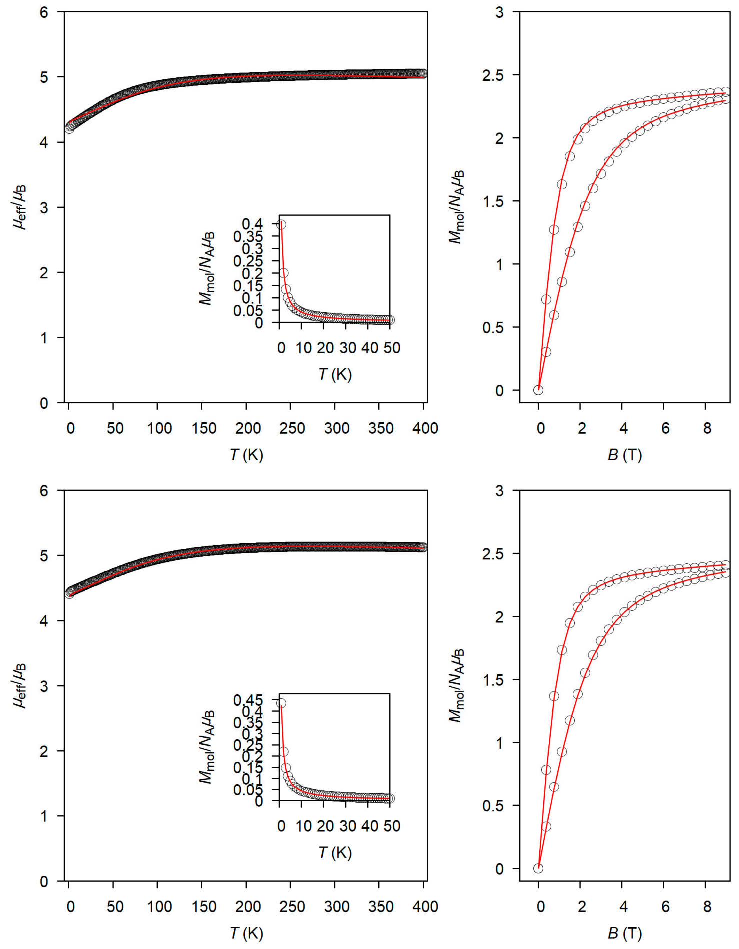

Figure S10. The analysis of the magnetic data calculated from CASSCF/NEVPT2 calculations with the fixed values of $\alpha\lambda$, Δ_{ax} and Δ_{rh}, and only values of α and λ were varied as listed in Table 2.

Table S1. The results of SHAPE calculation for 1-3.

	HP-6	PPY-6	OC-6	TPR-6	JPPY-6
**1**	29.075	14.123	11.893	3.761	18.120
**2**	29.532	18.007	7.650	9.801	22.143
**3**	28.787	19.702	6.352	10.110	23.913

HP-6, D_{6h} Hexagon, PPY-6, C_{5v}, Pentagonal pyramid, OC-6, O_h, Octahedron, TPR-6, D_{3h}, Trigonal prism, JPPY-6, C_{5v}, Johnson pentagonal pyramid J2

Table S2. Parameters of one-component Debye's model for 1 **derived** according to Eq. 2 in the main text.[a]

T/K	χ_S/(10^{-6}m^3 mol^{-1})	χ_T/(10^{-6}m^3 mol^{-1})	α	τ/(10^{-6}s)
2.30	2.824(0.093)	20.102(1.489)	0.338(0.028)	214113.070(43705.232)
2.40	2.742(0.085)	17.771(0.909)	0.299(0.025)	140613.414(19048.391)
2.50	2.691(0.075)	15.779(0.527)	0.244(0.022)	92726.025(7659.495)
2.60	2.626(0.074)	14.981(0.399)	0.225(0.021)	70423.439(4580.366)
2.70	2.527(0.065)	13.786(0.257)	0.178(0.018)	50510.415(2205.816)
2.80	2.448(0.059)	13.121(0.184)	0.153(0.015)	37793.546(1256.452)
2.90	2.359(0.053)	12.587(0.135)	0.135(0.013)	28386.359(743.132)
3.00	2.256(0.047)	12.107(0.101)	0.122(0.011)	21327.536(454.422)
3.10	2.154(0.040)	11.655(0.073)	0.108(0.009)	16054.018(272.475)
3.20	2.058(0.034)	11.257(0.055)	0.099(0.008)	12098.879(167.112)
3.30	1.977(0.030)	10.900(0.042)	0.088(0.007)	9159.992(106.271)
3.40	1.880(0.033)	10.607(0.041)	0.087(0.007)	7031.358(85.774)
3.50	1.829(0.028)	10.285(0.031)	0.075(0.006)	5355.918(53.432)
3.60	1.759(0.025)	9.978(0.025)	0.066(0.005)	4133.670(35.786)
3.70	1.700(0.026)	9.704(0.023)	0.060(0.005)	3227.284(27.715)
3.80	1.650(0.025)	9.456(0.021)	0.053(0.005)	2540.628(20.595)
3.90	1.605(0.024)	9.210(0.018)	0.047(0.004)	2018.770(15.022)
4.00	1.543(0.026)	8.991(0.018)	0.044(0.005)	1606.776(12.872)
4.10	1.510(0.024)	8.787(0.015)	0.040(0.004)	1304.647(9.221)
4.20	1.462(0.022)	8.589(0.013)	0.036(0.004)	1057.451(6.715)
4.30	1.390(0.029)	8.394(0.015)	0.036(0.005)	851.454(6.657)
4.40	1.342(0.025)	8.202(0.012)	0.032(0.004)	700.390(4.677)
4.50	1.330(0.025)	8.036(0.011)	0.027(0.004)	590.525(3.787)
4.60	1.295(0.021)	7.878(0.008)	0.026(0.003)	494.393(2.574)
4.70	1.257(0.019)	7.722(0.007)	0.025(0.003)	411.995(1.897)
4.80	1.176(0.026)	7.573(0.008)	0.028(0.004)	343.329(2.126)
4.90	1.248(0.026)	7.428(0.007)	0.014(0.004)	302.130(1.847)
5.00	1.102(0.026)	7.289(0.006)	0.026(0.003)	247.197(1.440)
5.10	1.178(0.025)	7.152(0.005)	0.013(0.003)	219.731(1.256)
5.20	1.208(0.039)	7.024(0.007)	0.006(0.005)	192.265(1.672)
5.30	1.168(0.037)	6.896(0.006)	0.007(0.004)	164.798(1.381)
5.40	0.989(0.033)	6.778(0.004)	0.021(0.003)	137.332(1.035)
5.50	1.072(0.037)	6.660(0.004)	0.009(0.003)	123.599(1.045)
5.60	1.118(0.048)	6.547(0.005)	0.003(0.004)	109.866(1.214)
5.70	1.077(0.052)	6.441(0.004)	0.005(0.004)	96.133(1.181)
5.80	0.932(0.050)	6.332(0.003)	0.014(0.004)	82.400(0.986)
5.90	0.706(0.098)	6.231(0.005)	0.025(0.006)	68.667(1.600)
6.10	0.709(0.109)	6.035(0.004)	0.019(0.006)	54.933(1.460)
6.40	0.851(0.178)	5.758(0.003)	0.009(0.008)	41.200(1.894)
6.50	1.240(0.216)	5.675(0.004)	0.000(0.010)	41.200(2.506)

[a] *the standard deviations of the fitted parameters are listed in the brackets.*

Table S3. Parameters of one-component Debye's model for 2 derived according to Eq. 2 in the main text.[a]

T/K	χ_S/(10^{-6}m^3 mol^{-1})	χ_T/(10^{-6}m^3 mol^{-1})	α	τ/(10^{-6}s)
1.90	1.719(0.050)	14.314(0.020)	0.140(0.004)	620.738(4.999)

2.00	1.560(0.070)	13.576(0.023)	0.142(0.005)	466.927(5.211)
2.10	1.450(0.101)	12.921(0.027)	0.142(0.007)	352.942(5.638)
2.20	1.252(0.139)	12.374(0.030)	0.145(0.009)	260.930(5.701)
2.30	1.106(0.213)	11.846(0.035)	0.145(0.012)	193.638(6.544)
2.40	0.765(0.297)	11.388(0.035)	0.155(0.015)	141.452(6.760)

[a] *the standard deviations of the fitted parameters are listed in the brackets.*

Table S4. Parameters of one-component Debye's model for 3 derived according to Eq. 2 in the main text.[a.]

T/K	χ_S/(10^{-6}m^3 mol^{-1})	χ_T/(10^{-6}m^3 mol^{-1})	α	τ/(10^{-6}s)
1.90	5.518(0.157)	15.417(0.034)	0.150(0.011)	265.050(7.469)
2.00	5.145(0.181)	14.645(0.035)	0.151(0.013)	237.584(7.919)
2.10	4.998(0.135)	13.929(0.025)	0.148(0.010)	219.731(5.755)
2.20	4.904(0.148)	13.443(0.026)	0.136(0.011)	207.371(6.055)
2.30	4.683(0.129)	12.806(0.021)	0.134(0.010)	190.891(5.063)
2.40	4.458(0.114)	12.286(0.017)	0.140(0.009)	173.038(4.220)
2.50	4.264(0.111)	11.770(0.015)	0.131(0.008)	160.678(3.913)
2.60	4.307(0.140)	11.306(0.019)	0.116(0.011)	155.185(4.954)
2.70	4.233(0.166)	10.860(0.021)	0.099(0.014)	144.199(5.543)
2.80	3.857(0.093)	10.496(0.010)	0.122(0.007)	126.346(2.822)
2.90	3.953(0.085)	10.129(0.009)	0.105(0.007)	123.599(2.617)
3.00	3.828(0.072)	9.776(0.007)	0.099(0.006)	113.986(2.115)
3.10	3.514(0.102)	9.471(0.008)	0.108(0.007)	100.253(2.645)
3.20	3.444(0.383)	9.168(0.028)	0.102(0.028)	93.386(9.551)
3.30	3.459(0.114)	8.869(0.008)	0.082(0.009)	87.893(2.725)
3.40	3.419(0.082)	8.608(0.005)	0.087(0.006)	82.400(1.917)
3.50	3.352(0.098)	8.360(0.006)	0.076(0.007)	76.906(2.173)
3.60	2.770(0.225)	8.127(0.009)	0.086(0.013)	60.427(3.720)
3.70	3.017(0.126)	7.912(0.005)	0.083(0.008)	60.427(2.278)
3.90	2.704(0.220)	7.499(0.006)	0.079(0.012)	48.067(3.161)
4.10	3.109(0.163)	7.144(0.004)	0.059(0.010)	46.694(2.605)
4.30	2.422(0.625)	6.809(0.008)	0.066(0.025)	31.587(6.148)
4.50	2.812(0.446)	6.494(0.005)	0.057(0.020)	30.214(4.913)
4.70	2.573(0.569)	6.229(0.004)	0.063(0.021)	23.347(4.818)

[a] *the standard deviations of the fitted parameters are listed in the brackets.*
